# Stress Conditions Triggering Mucoid Morphotype Variation in *Burkholderia* Species and Effect on Virulence in *Galleria mellonella* and Biofilm Formation In Vitro

**DOI:** 10.1371/journal.pone.0082522

**Published:** 2013-12-16

**Authors:** Inês N. Silva, Andreia C. Tavares, Ana S. Ferreira, Leonilde M. Moreira

**Affiliations:** 1 Department of Bioengineering, Instituto Superior Técnico, Lisbon University, Lisbon, Portugal; 2 Institute for Biotechnology and Bioengineering, Centre for Biological and Chemical Engineering, Instituto Superior Técnico, Lisbon, Portugal; Universidad Nacional de La Plata, Argentina

## Abstract

*Burkholderia cepacia* complex (*Bc*c) bacteria are opportunistic pathogens causing chronic respiratory infections particularly among cystic fibrosis patients. During these chronic infections, mucoid-to-nonmucoid morphotype variation occurs, with the two morphotypes exhibiting different phenotypic properties. Here we show that in vitro, the mucoid clinical isolate *Burkholderia multivorans* D2095 gives rise to stable nonmucoid variants in response to prolonged stationary phase, presence of antibiotics, and osmotic and oxidative stresses. Furthermore, in vitro colony morphotype variation within other members of the *Burkholderia* genus occurred in *Bc*c and non-*Bc*c strains, irrespectively of their clinical or environmental origin. Survival to starvation and iron limitation was comparable for the mucoid parental isolate and the respective nonmucoid variant, while susceptibility to antibiotics and to oxidative stress was increased in the nonmucoid variants. Acute infection of *Galleria mellonella* larvae showed that, in general, the nonmucoid variants were less virulent than the respective parental mucoid isolate, suggesting a role for the exopolysaccharide in virulence. In addition, most of the tested nonmucoid variants produced more biofilm biomass than their respective mucoid parental isolate. As biofilms are often associated with increased persistence of pathogens in the CF lungs and are an indicative of different cell-to-cell interactions, it is possible that the nonmucoid variants are better adapted to persist in this host environment.

## Introduction

The *Burkholderia cepacia* complex (*Bc*c) is a group of bacterial species that comprises opportunistic pathogens causing severe chronic infections in cystic fibrosis (CF) and immunocompromised patients. The respiratory tract of CF patients is predominantly colonized with *Pseudomonas aeruginosa*, *Staphylococcus aureus*, *Haemophilus influenza*, and, to a lesser extent, *Bc*c bacteria. Nevertheless, the infections caused by *Bc*c bacteria are extremely difficult to eradicate, leading in some cases to necrotizing pneumonia and septicemia [1], [2]. Within the CF lung, colonizing bacteria face an environment with high osmolarity, heterogeneous distribution of oxygen and nutrients, high concentration of antimicrobials, and constant challenge by the host immune defenses [3], [4]. These factors exert a selective pressure in colonizing bacteria and are thought to be the driving force of microevolution during their persistence in the CF lung. Phenotypic and genotypic variation within the CF lung have been well described in *P. aeruginosa* (reviewed in [5]) and is associated with mutator strains arising after long-term infection [6], [7]. Several studies have been undertaken to evaluate in vitro the effect of specific environmental stressors on the generation of *P. aeruginosa* variants in the CF lung. For example, under in vitro conditions of suboptimal nutrition, isolates of *P. aeruginosa* that produced rough lipopolysaccharide and were mucoid have emerged. These two phenotypes have been associated with long-term colonization of CF patients [8]. Similarly, prolonged incubation of nonmucoid *P. aeruginosa* in minimal medium with acetamide as the sole carbon source triggered the switch to the mucoid phenotype [9]. Mucoid variation of another *P. aeruginosa* isolate was also shown when cultivated in a chemostat system in a medium with high osmolarity or under carbon and nitrogen limitation [10]. Biofilm communities were also proven to be a source of self-generated diversity since *P. aeruginosa* was shown to undergo extensive genetic diversification during short-term growth in biofilms [11].

While analyzing the mucoid morphotype of more than 500 *Bc*c isolates from 100 chronically infected CF patients, Zlosnik and collaborators [12] reported thirteen cases of mucoid-to-nonmucoid transition of *Burkholderia multivorans*, *Burkholderia cenocepacia* and *Burkholderia vietnamiensis* sequential clonal isolates. These authors demonstrated that *Bc*c bacteria are also prone to phenotypic changes within the CF lung. Two such pairs of mucoid and nonmucoid variants from the species *B. cenocepacia* and *B. multivorans* showed numerous genotypic and phenotypic differences between the mucoid and the nonmucoid isolates [13], [14]. Besides lack of exopolysaccharide production, nonmucoid isolates formed more biofilm than the mucoid isolates and showed virulence attenuation in animal models of infection [13], [15]. A detailed characterization of the mucoid *B. multivorans* D2095 and the nonmucoid D2214 clonal isolates showed genetic variation caused by deletion and duplication of genes [13]. A general reduction of the level of expression of genes involved in several virulence factors in the nonmucoid D2214 isolate was also observed, namely motility, type-VI secretion, and hemolysin-type protein secretion. The D2214 nonmucoid isolate also showed a higher survival than the mucoid D2095 clonal isolate in carbon depleted medium for prolonged period of time, suggesting a higher ability to withstand stressful conditions [13]. The contribution of the mucoid and nonmucoid morphotypes to CF lung disease progression was evaluated by correlating the morphotype of the clinical isolates with lung function decline of the patients [16]. That study has shown the existence of an inverse correlation between the mucoid morphotype of the *Bc*c isolates and CF lung function deterioration. Despite the importance of the mucoid-to-nonmucoid phenotypic transition, both the triggering factors and the underlying molecular mechanisms are still unknown. The only exception are the antibiotics ciprofloxacin and ceftazidime, which have been demonstrated to trigger the mucoid-to-nonmucoid conversion in *B. cenocepacia* and *B. multivorans* clinical isolates in vitro [16]. Herein, we have evaluated the effect of multiple stress conditions on the rate of emergence of nonmucoid variants from the mucoid clinical isolate *B. multivorans* D2095. Stress conditions chosen are likely to be encountered by *Bc*c bacteria when colonizing the CF lung, as is the case of prolonged growth, presence of antibiotics, and osmotic, oxidative and nitrosative stresses. Moreover we have determined if other mucoid *Bc*c and non-*Bc*c isolates from clinical or environmental sources also yield nonmucoid variants. Lastly, we assessed whether these nonmucoid variants exhibited any advantage when exposed to conditions of nutrient limitation, biofilm formation, siderophore production, susceptibility to antimicrobial agents, and virulence towards *Galleria mellonella*.

## Materials and Methods

### Bacterial isolates and culture conditions

Bacteria used in this study are described in Table 1. *Burkholderia* strains were grown in LB or in EPS producing media: SM (12.5 g l^−1^ Na_2_HPO_4_; 3.0 g l^−1^ KH_2_PO_4_; 1.0 g l^−1^ K_2_SO_4_; 1.0 g l^−1^ NaCl; 0.2 g l^−1^ MgSO_4_.7H_2_O; 0.001 g l^−1^ CaCl_2_.2H_2_O; 0.001 g l^−1^ FeSO_4_.7H_2_O; 1.0 g l^−1^ casamino acids; 1.0 g l^−1^ yeast extract; 20 g l^−1^ mannitol), MM (2 g l^−1^ yeast extract; 20 g l^−1^ mannitol), and YEM (0.5 g l^−1^ yeast extract; 4 g l^−1^ mannitol) [12] at 30°C (non-*Bc*c strains) or 37°C (*Bc*c strains). SM is a minimal medium with a high carbon to nitrogen ratio stimulating exopolysaccharide production. Nevertheless, in the form of SM agar it is difficult to assess the mucoid phenotype of the *Bc*c colonies in the first 24–48 hrs. The best medium for mucoid morphotype assessment in the early hrs of colony growth (24–48 hrs) is YEM agar followed by MM agar. Liquid MM medium gives the highest yield of EPS production. Therefore, SM medium was used for growing cells under stressed or non-stressed conditions, YEM agar was used to distinguish between mucoid and nonmucoid morphotype, and MM liquid medium was used to quantify EPS production.

**Table 1:** Bacterial strains used in this study.

Isolate	Relevant characteristic(s)	EPS production (g/l)	Ciprofloxacin MIC (µg/ml)	Exposure [ciprofloxacin](µg/ml)	Source or reference
*B. multivorans* D2095	Cystic fibrosis clinical isolate, Canada; EPS^+^ (cepacian)^§^	3.7±0.1	4	10	[13]
*B. multivorans* D2214	Cystic fibrosis clinical isolate, Canada; EPS^−^	0.0	ND	-	[13]
*B. multivorans* ATCC 17616	Soil isolate, USA; EPS^+^(cepacian)^ §^	0.3±0.1	1	2.5	[33]
*B. cepacia* IST408	Cystic fibrosis clinical isolate, Portugal; EPS^+^ (cepacian)^ §^	1.2±0.5	1	2.5	[34]
*B. cepacia* ATCC 17759	Soil isolate, Trinidad; EPS^+#^	ND	1	2.5	[35]
*B. cenocepacia* J415	Cystic fibrosis clinical isolate, UK; EPS^+#^	ND	2	5	[36]
*B. stabilis* LMG14086	Respirator, UK; EPS^+#^	ND	1	2.5	[37]
*B. stabilis* LMG18888	Human blood, Belgium; EPS^+#^	0.2±0.1	1	2.5	[38]
*B. vietnamiensis* PC259	Cystic fibrosis clinical isolate, USA; EPS^+#^	ND	1	2.5	[39]
*B. ambifaria* AMMD	Root-colonizing bacterium, USA; EPS^+^ (cepacian)^ §^	ND	1	2.5	[40]
*B. ambifaria* CEP0958	Cystic fibrosis clinical isolate, Australia; EPS^+#^	3.9± 0.3	1	2.5	[40]
*B. dolosa* CEP0743	Cystic fibrosis clinical isolate, Canada; EPS^+#^	1.2±0.4	2	5	D. P. Speert
*B. anthina* J2552	Soil isolate, UK; EPS^+#^	ND	1	2.5	[41]
*B. anthina* FC0974	Cystic fibrosis clinical isolate, Canada; EPS^+#^	0.4±0.2	1	2.5	D. P. Speert
*B. phymatum* STM815	Soil isolate, French Guiana; nitrogen fixation; EPS^+^ (cepacian)^ §^	1.9±0.2	6^*^	15	[42]
*B. xenovorans* LB400	Soil isolate, USA; degradation of polychlorinated biphenyl compounds; EPS^+^ (cepacian)^ §^	5.2±0.2	6^*^	15	[43]

ND, not determined; ^*^MIC determined at 30°C; ^§^ the EPS produced by this strain in MM medium is cepacian as determined by condensed-phase infrared spectroscopy [44]; ^#^ the nature of this EPS was not determined; EPS^+^, producer of exopolysaccharide; EPS^−^, non EPS producer.

### Bacterial genotyping

Genomic DNA preparation and pulsed-field gel electrophoresis (PFGE) were performed as described previously [17]. Prior to PFGE, immobilized DNA was digested with 20 U of SpeI restriction endonuclease before loading into a 1% (wt vol^−1^) agarose gel in 0.5 x-TBE buffer. A Gene Navigator apparatus (Pharmacia-LKB, Sweden) was used at 200 V with 5-120 seconds pulse times for 22 hrs.

### DNA manipulation techniques

Genomic DNA was extracted and purified using the DNeasy blood and tissue kit (Qiagen) following the manufactureŕs recommendations. PCR amplification of genes homologous to *B. multivorans* ATCC 17616 Bmul_4804, Bmul_4809, Bmul_4876, and Bmul_4781 was done under the following conditions: 5 min at 95°C; 34 cycles of 30 seconds at 95°C, 45 seconds at 57°C and 1 min at 72°C; followed by an extension step at 72°C for 7 min. Primer sequences are shown in Table S1. Amplification products were separated by 0.8% (wt vol^−1^) agarose gel electrophoresis.

### Switching of colony morphology during laboratory stress conditions

Several culture conditions were examined to assess the emergence of morphotype (mucoid vs. nonmucoid) variation in *Burkholderia* isolates under study. To test prolonged incubation, triplicates of *Burkholderia* cultures were inoculated in 3-ml of SM medium (OD_640nm_ of 0.1) and maintained statically at 37°C for 21 days. Aliquots were removed at day 0, 7, 14, and 21, serially diluted, spread onto the surface of YEM agar plates, and incubated at 37°C for 2 days. Plates were examined with respect to the number of colony forming units (c.f.u.) and the proportion of the mucoid and the nonmucoid morphotypes. Colonies showing nonmucoid appearance were picked to MM solid medium and incubated at 37°C for 2 days. After a 2^nd^ passage, exopolysaccharide production in liquid medium and genotyping of two representative nonmucoid colonies of *B. multivorans* D2095 and one of *B. cepacia* IST408, *B. multivorans* ATCC 17616, *B. dolosa* CEP0743, *B. anthina* FC0974, *B. phymatum* STM815 and *B. xenovorans* LB400 was performed.

Other tested conditions were statical growth in liquid SM for 21 days at 4°C, 22°C, or 42°C, or at 37°C for 7 days in the presence of NaCl (10 mg ml^−1^), sodium hypochlorite (0.1% vol vol^−1^), S-nitrosoglutathione (2.5 mM), the antibiotics ciprofloxacin (10 µg ml^−1^), ceftazidime (40 µg ml^−1^), amikacin (195 µg ml^−1^), and kanamycin (50 µg ml^−1^), and microaerophilia (generators GENbox microaer, BioMérieux contained in 2 L sealed jars). Antibiotics minimal inhibitory concentration (MIC) were determined by broth serial microdilution [18]. Under each of these stress conditions where morphotype variation had occurred, one nonmucoid colony derived from each mucoid parental isolate was retained for further analysis.

To determine whether mucoid-to-nonmucoid morphotype transition was reversible, one nonmucoid variant was recovered from each condition and each mucoid parental isolate, and incubated in SM medium for 21 days statically at 37°C. Aliquots at different time points were taken, spread onto YEM agar plates and colony morphotype was assessed as described above.

### Exopolysaccharide production

The amount of EPS produced was assessed based on the dry-weight of the ethanol-precipitated polysaccharide recovered from 100-ml cultures of the different strains grown in liquid MM over 6 days at 30°C with agitation, as described before [19]. Results are the means of three independent experiments.

### Long-term nutrient starvation

Bacterial cells grown overnight in LB liquid medium were harvested, washed twice with NaCl 0.9% (wt vol^−1^) and inoculated in duplicate in 50 ml of M63 minimal medium (2 g l^−1^ (NH4)2SO4, 13.6 g l^−1^ KH2PO4, 0.5 mg l^−1^ FeSO4 7H2O (pH 7), supplemented with 0.2 g l^−1^ MgSO_4_.7H_2_O) [20] without a carbon source to an OD_640nm_ of 1.0. Cultures were grown for 28 days at 37°C and 250 rpm orbital agitation. Samples were taken at day 0, 15 and 28, adequately diluted and plated onto the surface of YEM solid medium. C.f.u. were quantified and percentages of survival were determined.

### Antimicrobial susceptibility

Antimicrobial susceptibility was assessed based on the agar disc diffusion method [21] using paper discs containing imipenem (10 µg), aztreonam (30 µg), piperacillin (75 µg) plus tazobactam (10 µg), ciprofloxacin (5 µg), ceftazidime (30 µg) and amikacin (30 µg). The discs were applied onto the surface of Mueller-Hinton (Difco Laboratories) agar plates previously inoculated with 100 µl of bacterial cultures grown for 5 hrs in SM, at 37°C and diluted to OD_640nm_ 0.1. Growth inhibition diameter was measured after 24 hrs incubation at 37°C. For zone inhibition assays, 100 µl of each culture with OD_640nm_ 0.1 were spread onto SM plates. Sterile paper discs (6 mm diameter) were placed onto the agar surface. A total of 10 µl of H_2_O_2_ (30% vol vol^−1^) was pipetted onto separate discs. The plates were incubated for 24 hrs at 37°C and the diameter of the growth inhibition zone was measured. Results are the means of three independent experiments.

### Biofilm formation

Biofilm formation assays were performed as previously described [19]. Bacteria were grown in SM medium at 37°C to mid-exponential phase, diluted to OD_640nm_ 0.05, and 200 µl of these cell suspensions were used to inoculate the wells of a 96-well polystyrene microtitre plate. Plates were incubated at 37°C statically for 48 hrs. The biofilm was stained with crystal violet solution, followed by dye solubilization with ethanol and measurement of the solution’s A_590nm_ using a microplate reader. Results are the means of five independent experiments.

### Siderophore production

The secretion of siderophores was measured by the CAS assay [22] with slight modifications. Briefly, overnight cultures were grown as previously described, diluted to an OD_640nm_ of 1.0 and 5 µl spots were inoculated onto the surface of CAS-MM agar plates. Plates were incubated at 37°C and siderophore production was assessed by measuring the diameter of the yellow haloes after 48 hrs of incubation.

### Virulence determination in *Galleria mellonella*

Killing assays were performed as described before [23]. For this purpose larvae were injected with cell suspensions containing a total c.f.u. ranging from 1×10^4^ to 1×10^7^ in 10 mM MgSO_4_ with 1.2 mg ml^−1^ ampicillin, and incubated at 37°C. Survival rate was evaluated during the following 7 days post-infection. As a negative control, 10 mM MgSO_4_ with 1.2 mg ml^−1^ ampicillin was used. Triplicates of ten larvae were used in each experiment.

### Statistical analysis

All quantitative data was obtained from at least three independent assays. Standard deviation was used to calculate error bars. The *t*-test to determine *P*-values and the Kaplan Meier survival curves were performed using GraphPad Prism 5.0 software. Differences were considered to be statistically significant if the *P*-value was lower than 0.05.

## Results

### Conversion of the mucoid-to-nonmucoid morphotype occurred during prolonged stationary phase in vitro and was not reversible

**Figure 1:**
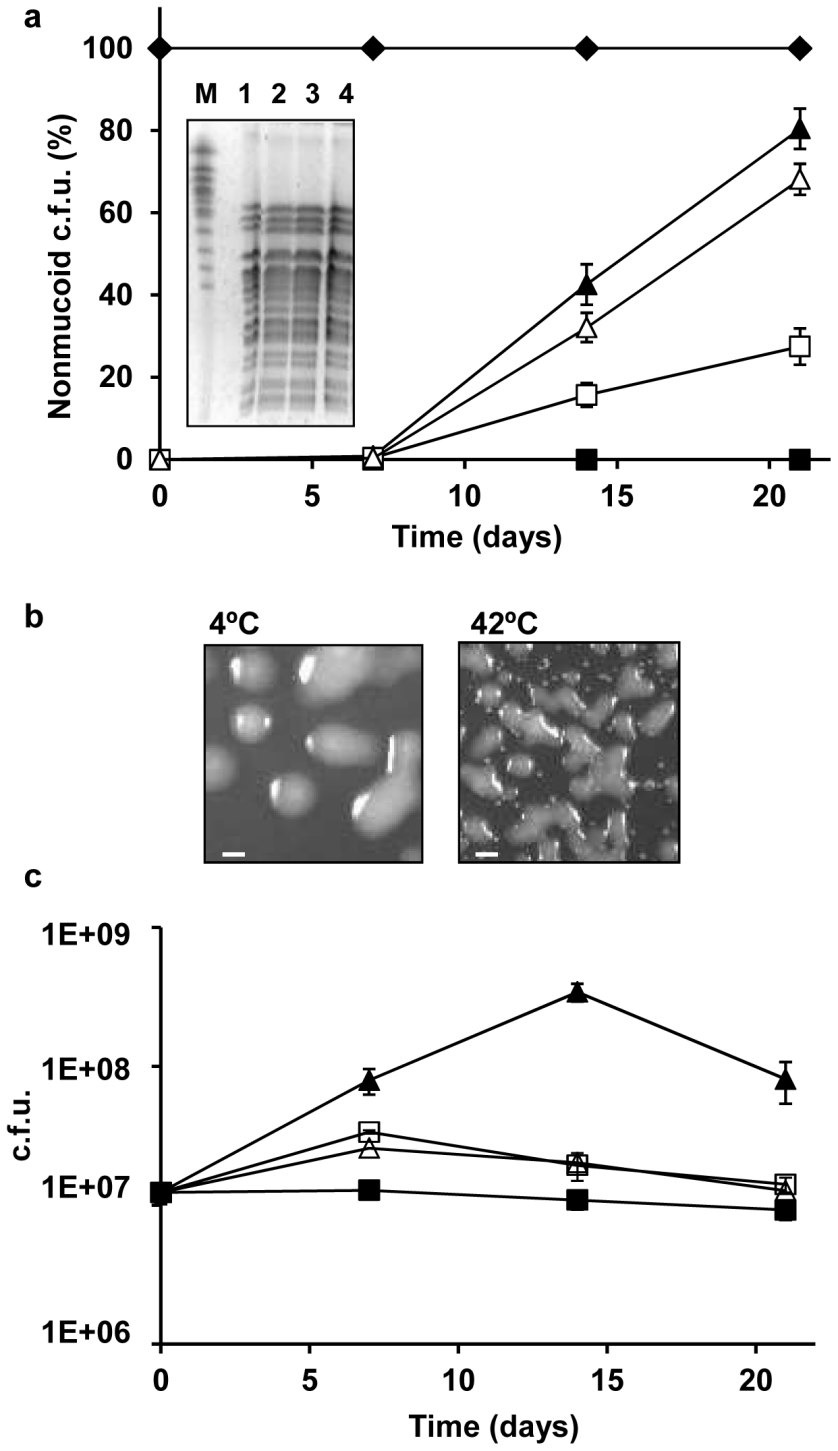
Colony morphotype variation of *B. multivorans* D2095 and D2214 during in vitro prolonged stationary phase. A single colony of each isolate was cultured into SM liquid medium and cells plated onto YEM agar after 7, 14, and 21 days of static incubation at different temperatures in air. (a) Frequency of the nonmucoid morphotype of *B. multivorans* D2214 (♦) incubated at 37°C and *B. multivorans* D2095 at 4°C (▪), 22°C (▴), 37°C (□) and 42°C (▵). The panel inside this figure shows the SpeI restriction fragments of the genomic DNA from D2095 (1) and nonmucoid variants obtained at: 22°C (2); 37°C (3) and 42°C (4) separated by PFGE. The chromosomes of *Saccharomyces cerevisiae* strain YPH80 (Sigma) were run as standard (M). (b) Colony morphotypes of *B. multivorans* D2095 cultures after incubation for 21 days at 4°C (left panel) or 42°C (right panel) and plating onto YEM agar and incubation for 3 days at 37°C (bar, 5 mm). (c) Total colony count during the time of the experiment described in (a). Error bars show SD. The data are based on mean values from the results of three independent cell cultivations.

To understand whether colony mucoid morphotype variation takes place in vitro, the sequential isolates *B. multivorans* D2095 and D2214 from a cystic fibrosis patient were used. *B. multivorans* D2095 was recovered 13-years after the first *B. multivorans* isolation in that patient and its colonies, as well as the ones of all the previous isolates, are mucoid in SM medium [13]. The clonal *B. multivorans* D2214, recovered six-months later than D2095, shows a nonmucoid morphotype, corroborated by the down-regulated expression of the *bce* genes directing cepacian biosynthesis [13]. To evaluate mucoid morphotype variation, isolates D2095 and D2214 were inoculated into SM medium at 37°C statically for 3 weeks. During this period, cultures were sampled and culture aliquots were plated onto YEM agar medium at different time points and the mucoid phenotype was assessed. The behavior of the two isolates was distinct. While the nonmucoid D2214 isolate never gave rise to mucoid colonies, the percentage of nonmucoid colonies arising from the mucoid D2095, increased over time (Fig. 1a). After 3 weeks of prolonged incubation, the nonmucoid colonies derived from D2095 accounted for approximately 27% of the total scored colonies.

To evaluate if temperatures above and below the optimal for growth could potentiate the mucoid morphotype variation observed during prolonged stationary phase, the mucoid isolate D2095 was incubated at 4, 22 and 42°C under the conditions described above. At 4°C, no morphotype variation was observed during the assay time period, with all colonies displaying the mucoid phenotype (Figs. 1a and 1b). At this temperature, a slight reduction of the total c.f.u. was registered all over the tested period (Fig. 1c). Contrastingly, at 22 and 42°C a strong increase in the percentage of nonmucoid colonies was observed, reaching approximately 80 and 70% of the total number of colonies, respectively (Figs. 1a and 1b). Total c.f.u. count at 42°C showed a pattern similar to the observed at 37°C, with an increase during the first week followed by a reduction until day 21 (Fig. 1c). At 22°C the c.f.u. count increased sharply until day 14, followed an abrupt decrease (Fig. 1c).

To assess if the nonmucoid variants from D2095, obtained at 22, 37 and 42°C, had detectable genomic alterations, we performed total DNA digestion with SpeI restriction endonuclease followed by PFGE analysis. The pattern of the DNA bands obtained did not allow the differentiation of the three variants from the mucoid parental D2095 isolate (Fig. 1a, inside panel), suggesting that no detectable changes had occurred in their genome structure. The lack of exopolysaccharide production in a total of 4 representative nonmucoid variants was confirmed by growth in liquid MM medium followed by ethanol precipitation of the cell-free supernatant (data not shown).

To determine whether the conversion of the mucoid-to-nonmucoid morphotype was reversible, three nonmucoid colonies derived from *B. multivorans* D2095 were studied. The 3 nonmucoid variants were grown in SM medium for 21 days. After analysis of the morphotype of more than 3000 c.f.u. from each original culture at days 7, 14 and 21, no mucoid colonies could be detected.

### Inhibitory concentration of antibiotics and oxidative and osmotic stresses also triggered mucoid morphotype variation

**Figure 2:**
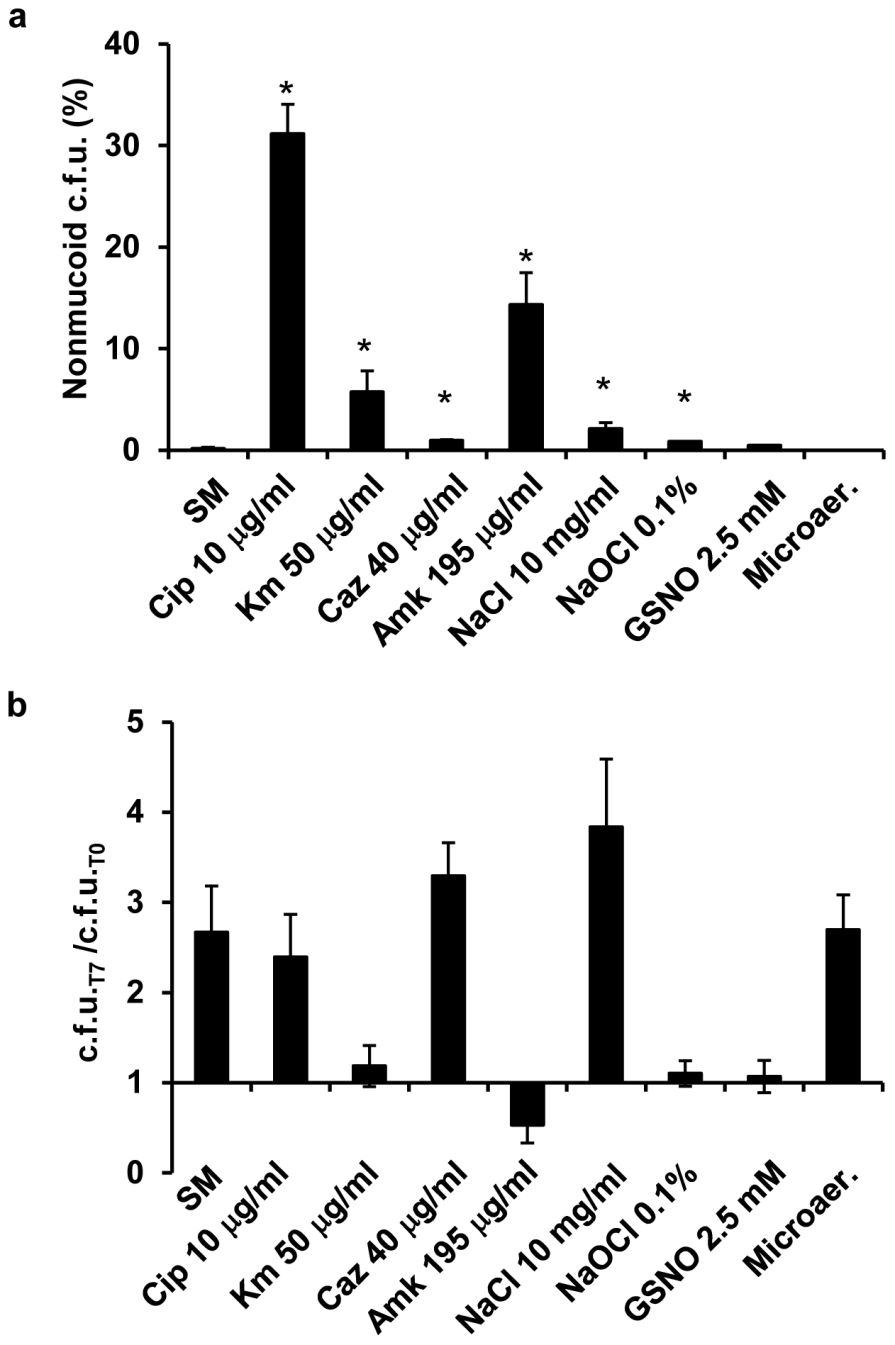
Stress conditions trigger mucoid morphotype variation. (a) Frequency of nonmucoid morphotype of *B. multivorans* D2095 incubated at 37°C for 7 days in SM liquid medium under the indicated stress conditions. (b) Ratio of c.f.u. obtained by dividing the number of c.f.u. at day 7 by the initial number of c.f.u.. Cip, ciprofloxacin; Km, kanamycin; Caz, ceftazidime; Amk, amikacin; GSNO, S-nitrosoglutathione; Microaer, microaerophilic conditions. Error bars show SD. The data are based on mean values from the results of three independent cell cultivations. The *t*-test was performed using GraphPad Prism 5.0 software. A *P*-value of <0.05 was considered significant compared with the condition of SM medium only (*).

Additional stress conditions were imposed to *B. multivorans* D2095 and the rates of mucoid-to-nonmucoid variation were evaluated. To discard the possible contribution of prolonged stationary phase in triggering mucoid variation, the effect of the other stresses was considered positive if the percentage of nonmucoid colonies isolated on the 7^th^ day of incubation was higher than that obtained with SM liquid medium alone. The stress conditions here imposed to *B. multivorans* D2095 were: growth in the presence of inhibitory concentration of some clinically relevant antibiotics such as ciprofloxacin (MIC 4 µg ml^−1^), ceftazidime (MIC 16 µg ml^−1^), amikacin (MIC 78 µg ml^−1^) and kanamycin (MIC 20 µg ml^−1^); growth in the presence of the osmotic stress agent sodium chloride, the oxidative stress agent sodium hypochlorite, the nitrosative stress agent S-nitrosoglutathione, and growth under microaerophilic conditions. Concentrations of the stress-causing agents were the lowest at which the mucoid morphotype variation was observed. For consistency, antibiotics concentration chosen were 2.5 times the MIC, which reflects a concentration able to stimulate the mucoid-to-nonmucoid transition and at the same time allows a large number of cells to remain viable. After incubation for 7 days in the presence of the tested antibiotics, the percentage of nonmucoid colonies ranged 1–30%. Ciprofloxacin induced the highest percentage of morphotype variation (Fig. 2a). Increased salt concentration, sodium hypochlorite, and S-nitrosoglutathione also triggered mucoid morphotype switch, with frequencies ranging 0.4–2.1%. Results obtained with S-nitrosoglutathione were statistically not significant (Fig. 2a). Microaerophilic conditions, although still promoting cell growth (Fig. 2b), did not stimulate morphotype variation within the tested period of time. The genomic DNA of one nonmucoid variant obtained under each tested stress condition was analyzed by PFGE. All the nonmucoid variants examined exhibited a DNA restriction pattern undistinguishable from the one of the mucoid parental *B. multivorans* D2095 isolate (data not shown).

### A DNA region absent from the genome of the nonmucoid D2214 isolate is present in mucoid D2095 and in its clonal nonmucoid variantśgenome

Previous genomic DNA hybridization of *B. multivorans* D2095 and D2214 isolates against DNA probes from *B. multivorans* ATCC 17616 present in a GeneChip array identified 52 genes that seemed to be absent from D2214 isolate but present in D2095 [13]. As the majority of these genes are clustered together in chromosome 2 of *B. multivorans* ATCC 17616, this suggests that a deletion event had taken place in D2214 isolate [13]. Although there is no evidence of any kind that those missing genes are involved in the mucoid-to-nonmucoid transition, we have examined the presence of a few of those genes in the *B. multivorans* D2095 nonmucoid variants obtained under all tested stress conditions by PCR amplification. The chosen genes, located at the beginning and at the end of the homologous *B. multivorans* ATCC 17616 genomic region, are homologous to genes Bmul_4804 (putative hemerythrin-like metal-binding protein), Bmul_4809 (putative MarR family transcriptional regulator), Bmul_4781 (putative 4,5-dihydroxyphthalate decarboxylase) and Bmul_4786 (putative ABC transporter). Results confirmed the absence of the 4 tested genes from *B. multivorans* D2214 genome, but their presence in the mucoid *B. multivorans* D2095 isolate, as well as in all the nonmucoid variants derived from the D2095 mucoid isolate (data not shown). This data, together with the undistinguishable PFGE restriction profiles of the nonmucoid variants and the parental mucoid D2095 isolate, suggest that the DNA region deleted in the D2214 isolate is not involved in the mucoid morphotype variation observed in the nonmucoid variants obtained in the present work.

### Prolonged stationary phase and ciprofloxacin triggered mucoid morphotype variation in *Bc*c and non-*Bc*c isolates

**Figure 3:**
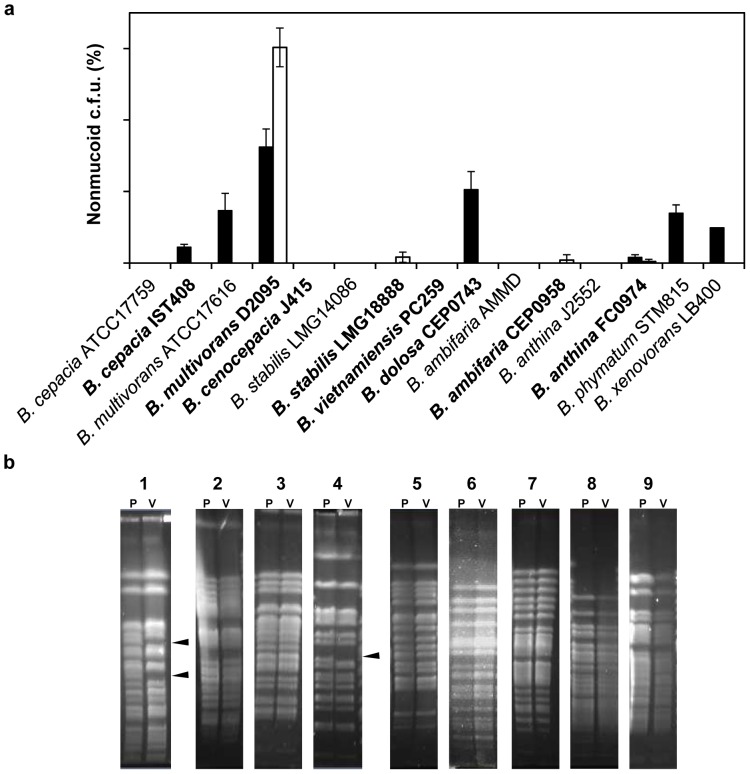
Morphotype variation among clinical and environmental isolates of *Burkholderia*. (a) The frequency of the nonmucoid morphotype was determined under prolonged stationary phase for 21 days (black bars) and in the presence of 2.5 times the MIC of ciprofloxacin of each mucoid isolate (white bars) after 7 days of static incubation at 37°C for *Bc*c isolates or 30°C for non-*Bc*c isolates. The data are based on mean values from the results of three independent cell cultivations. Error bars show SD. Clinical isolates are depicted in bold. (b) PFGE separation of the SpeI restriction fragments of the genomic DNA from the mucoid parental isolate (P) and the nonmucoid variant (V) of: *B. cepacia* IST408 (1); *B. multivorans* ATCC 17616 (2) and D2095 (3); *B. stabilis* LMG18888 (4); *B. dolosa* CEP0743 (5); *B. ambifaria* CEP0958 (6); *B. anthina* FC0974 (7); *B. phymatum* STM815 (8); and *B. xenovorans* LB400 (9). Arrowheads indicate differences in banding patterns.

Mucoid-to-nonmucoid morphotype variation under prolonged incubation conditions or in the presence of ciprofloxacin was also evaluated in several other mucoid *Burkholderia* isolates (Table 1). These isolates were chosen due to their ability to produce EPS in SM, YEM and MM media. These included non-*Bc*c environmental isolates (*B. phymatum* STM815 and *B. xenovorans* LB400), *Bc*c clinical isolates (*B. multivorans* D2095; *B. cepacia* IST408; *B. cenocepacia* J415; *B. stabilis* LMG18888; *B. vietnamiensis* PC259; *B. ambifaria* CEP0958; *B. dolosa* CEP0743; and *B. anthina* FC0974), and *Bc*c environmental isolates (*B. multivorans* ATCC 17616; *B. cepacia* ATCC 17759; *B. stabilis* LMG14086; *B. ambifaria* AMMD; and *B. anthina* J2552). Mucoid isolates were incubated in SM liquid medium statically at 37°C for 21 days. The percentage of nonmucoid colonies was evaluated. Prolonged growth was shown to trigger mucoid-to-nonmucoid variation among clinical and environmental *Bc*c strains - *B. cepacia* IST408, *B. multivorans* ATCC 17616 and D2095, *B. dolosa* CEP0743 and *B. anthina* FC0974 - and among environmental non-*Bc*c strains - *B. phymatum* STM815 and *B. xenovorans* LB400. The frequencies of mucoid-to-nonmucoid variation ranged 1–16% (Fig. 3a). Exposure of mucoid bacteria to concentrations of ciprofloxacin 2.5 times the MICs (Table 1) for 7 days resulted in the isolation of nonmucoid colonies from clinical isolates only, with *B. multivorans* D2095 isolate exhibiting a frequency of approximately 30%. *B. stabilis* LMG18888, *B. ambifaria* CEP0958, and *B. anthina* FC0974 frequencies of mucoid-to-nonmucoid variation ranged 0.2–0.9% (Fig. 3a). The ciprofloxacin concentration of 2.5 times the MIC used for *B. phymatum* STM815 and *B. xenovorans* LB400 did not allow recovery of viable cells. Therefore, these strains were exposed to the sub-inhibitory concentration of 3 µg ml^−1^ ciprofloxacin. This ciprofloxacin concentration had no effect on cell growth and did not induce morphotype variation. From *B. cepacia* ATCC 17759, *B. cenocepacia* J415, *B. stabilis* LMG14086, *B. vietnamiensis* PC259, *B. ambifaria* AMMD, and *B. anthina* J2552 we were unable to isolate nonmucoid variants under both tested conditions.

A total of 11 nonmucoid variants obtained under prolonged incubation or when using 2.5 times the MIC of ciprofloxacin, together with their respective mucoid parental isolates, were analyzed for exopolysaccharide production. Results showed that none of the variants produced EPS after 6 days of incubation in MM medium (data not shown). PFGE SpeI-restricted profiles of the nonmucoid variants confirmed they are indistinguishable from their respective mucoid parental isolates (Fig. 3b). *B. cepacia* IST408 and *B. stabilis* LMG18888 are an exception, since genomic re-arrangements are evident for the respective nonmucoid variants. Genomic re-arrangements in a *B. cenocepacia* CF isolate were shown to occur due to movement of insertion sequences caused by exposure to oxidative stress [24].

The phenotypic stability of the nonmucoid variants obtained in the presence of ciprofloxacin was tested by assessing the morphotype of a representative colony of each isolate after 7 days of incubation in SM liquid media at 37°C. Nonmucoid variants obtained under prolonged growth were incubated in the same medium for 21 days. Aliquots of each culture were taken at day 7 for the ciprofloxacin-induced variants, and at day 7, 14 and 21 for the nutrient starvation-induced variants, and plated onto YEM plates. After 3 days of incubation the morphotype of more than 3000 colonies of each isolate at each time point were analysed. All colonies were nonmucoid.

### Mucoid strains showed increased virulence in *Galleria mellonella*

**Figure 4:**
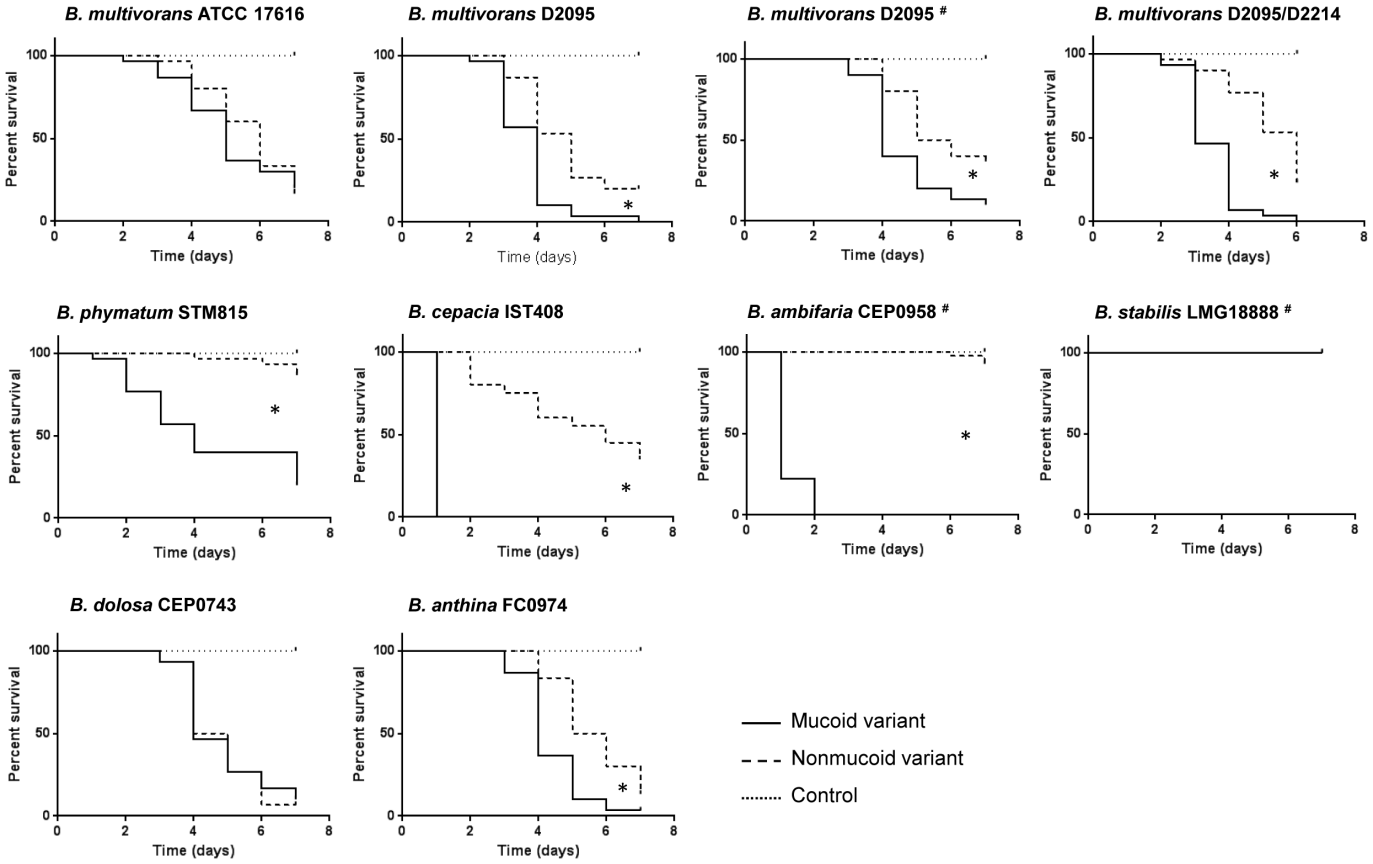
Survival of *Galleria mellonella* larvae inoculated with mucoid isolates and nonmucoid variants of *Burkholderia*. Triplicate groups of 10 larvae were inoculated with the mucoid parental isolate or the nonmucoid variants obtained during prolonged stationary phase or stress by ciprofloxacin (indicated by the symbol #). The control experiment without bacteria is also represented. Larvae were injected with approximately 1×10^5^ *Burkholderia* cells with the exceptions of *B. phymatum* STM815 and *B. stabilis* LMG18888 with approximately 1×10^7^ cells and *B. cepacia* IST408 and *B. ambifaria* CEP0958 with 1×10^4^ cells. Survival rates were determined for 7 days. A *P*-value of <0.05 was considered significant compared with the respective mucoid parental isolate (*).

Most of the mucoid *Burkholderia* isolates under study and respective nonmucoid variants were tested for virulence in *G. mellonella*. Since different growth rates may affect the number of bacteria following infection of host cells, we evaluated the growth rate of each mucoid and nonmucoid pair. Under the tested conditions (SM medium at 37°C for 32 hrs) no significant differences in the specific growth rate were observed for each variant pair (data not shown). The minimal number of c.f.u. that still resulted in larvae killing was also optimized and it was approximately 1×10^4^ for *B. cepacia* IST408 and *B. ambifaria* CEP0958; 1×10^5^ for *B. multivorans* ATCC 17616 and D2095, *B. dolosa* CEP0743, and *B. anthina* FC0974; and 1×10^7^ for *B. phymatum* STM815. *B. stabilis* LMG18888 was unable to kill larvae even when 1×10^7^ c.f.u. were used. Larvae survival was assessed over a period of 7 days and the results are shown in Fig. 4. Four nonmucoid variants derived from *B. multivorans* D2095 obtained under different stress conditions and the nonmucoid clinical isolate D2214 were compared with the mucoid D2095 isolate concerning their ability to kill larvae. The survival curves showed a statistically significant virulence attenuation of all the D2095 nonmucoid variants (for conciseness only two of them are shown in Fig. 4). Similarly, the nonmucoid clinical isolate *B. multivorans* D2214 induced lower larvae mortality rate as previously shown (Fig. 4) [13]. Among the remaining *Bc*c parental isolates examined, only one nonmucoid variant from each mucoid isolate was tested. Data showed virulence attenuation of the nonmucoid variant of *B. phymatum* STM815, *B. cepacia* IST408, *B. ambifaria* CEP0958 and *B. anthina* FC0974. No statistically significant differences in virulence were observed between the mucoid and nonmucoid variants of *B. multivorans* ATCC 17616 and *B. dolosa* CEP0743. The virulence of *B. stabilis* LMG18888 was inconclusive since no larvae killing was observed (Fig. 4).

**Figure 5:**
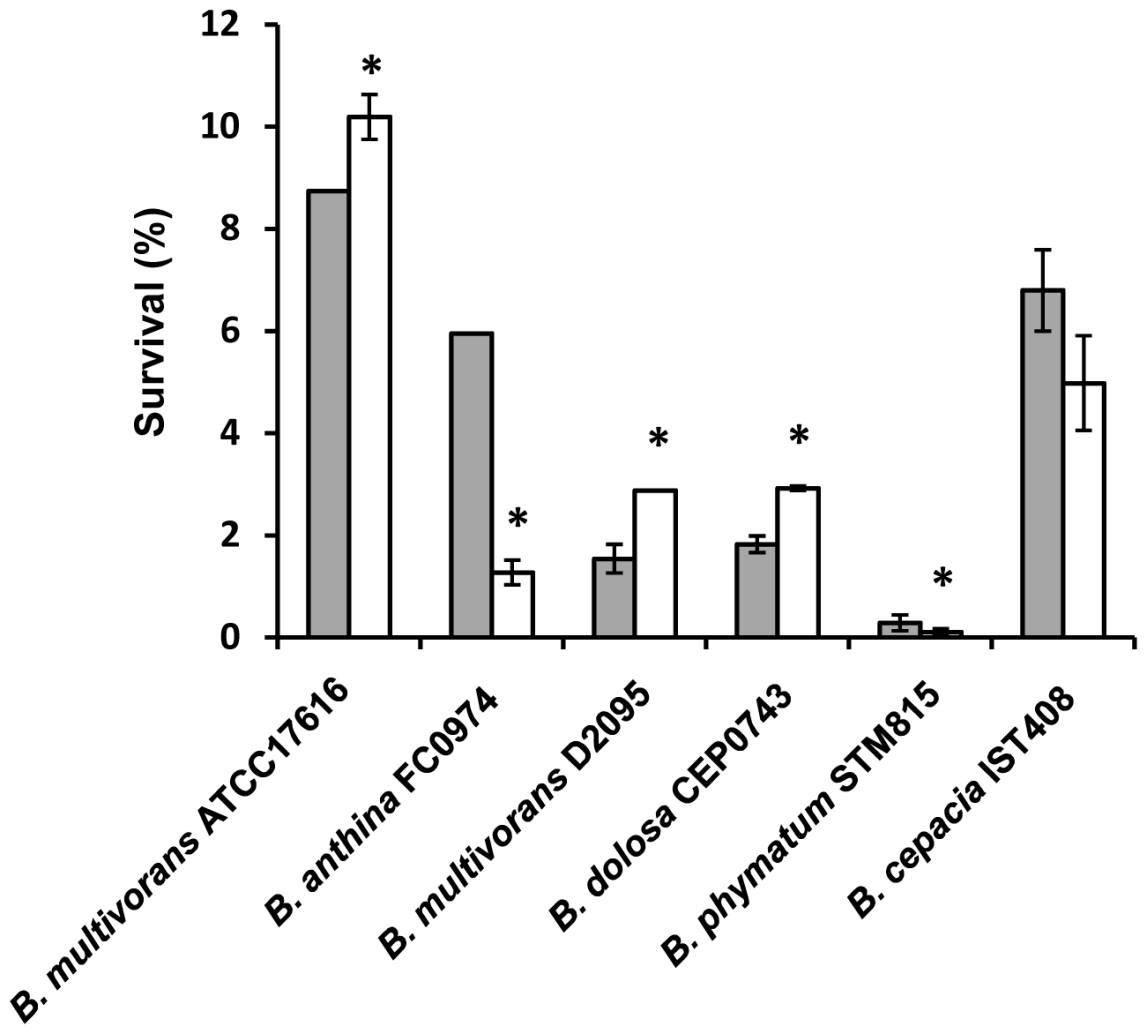
Ability of *Burkholderia* isolates and their respective nonmucoid variants to survive in M63 minimal medium without a carbon source. Percentages of survival at day 28 for mucoid parental strains (grey bars) and nonmucoid variants (white bars) are shown. The data are means ± SD from the results of at least three independent experiments. A *P*-value of <0.05 was considered significant compared with the respective mucoid parental isolate (*).

To determine if the mucoid and the nonmucoid variants have different survival ability under nutrient starvation, each mucoid isolate and the respective nonmucoid variant previously obtained were grown aerobically in minimal medium without carbon source for 28 days, at 37°C. Percentages of survival at the 28^th^ day were below 11% for all the tested isolates (Fig. 5). Although slight differences could be observed for each mucoid/nonmucoid pair of isolates, no correlation could be established between morphotype and ability to survive nutrient starvation. Survival to prolonged nutrient limitation seemed to be higher for the nonmucoid isolates of *B. multivorans* ATCC 17616 and D2095 and *B. dolosa* CEP0743, but lower for *B. anthina* FC0974 and *B. phymatum* STM815.

### In general, nonmucoid variants were more susceptible against antimicrobials

Six of the clinical mucoid isolates and the respective nonmucoid variants obtained under prolonged incubation (except for *B. ambifaria* CEP0958 and *B. stabilis* LMG18888 which were obtained in the presence of ciprofloxacin) were tested for susceptibility to several antibiotics as shown in Table 2. In general, the nonmucoid variants were more susceptible to the tested antibiotics. This is particularly evident for the nonmucoid variants of *B. multivorans* D2095 and *B. cepacia* IST408 which were more susceptible to all the antibiotics tested. In contrast, the nonmucoid clinical isolate *B. multivorans* D2214 was more susceptible than the mucoid D2095 to three antibiotics only (Table 2). *B. ambifaria* CEP0958 nonmucoid variant was more susceptible to four out of the six antibiotics tested. The remaining mucoid/nonmucoid pairs displayed a similar susceptibility to the antibiotics tested. Besides ciprofloxacin resistance, the nonmucoid variants of *B. ambifaria* CEP0958 and *B. stabilis* LMG18888 were the only ones showing increased resistance to imipenem and aztreonam, respectively. Piperacillin/tazobactam was the antibiotic formulation to which more nonmucoid variants displayed a higher susceptibility than the respective mucoid parental isolate.

**Table 2:** Antimicrobial susceptibility of mucoid isolates and nonmucoid variants.

Isolate	Growth inhibition (mm)						
	Amikacin	Piperacillin+ tazobactam	Aztreoname	Ciprofloxacin	Imipenem	Ceftazidime	H_2_O_2_
*B. multivorans* D2095	13.8±0.5	21±1	0±0	17.8±0.5	14.5±0.6	23.5±1.3	60.3±0.5
D2095 nonmucoid	22.3±1*	41.7±0.6*	29.5±0.6*	21.5±0.6*	16±0.8*	35.8±1*	54.5±0.6*
*B. multivorans* D2214	14.3±0.6	23.3±0.6*	0±0	21.0±1.4*	13.7±0.6	29.7±0.6*	59.0±1.4
*B. dolosa* CEP0743	11.8±0.5	12±0.8	0±0	17±0.8	0±0	14.5±0.6	54.8±1.0
CEP0743 nonmucoid	14.5±1.3*	10.7±0.6	0±0	16.8±0.5	0±0	12.8±0.5	53.3±1.3
*B. cepacia* IST408	20.8±0.5	30.3±0.6	20.3±1.3	28.8±0.5	21.8±1.3	27.3±0.5	54.8±1.0
IST408 nonmucoid	34±1.0*	39.3±1.2*	32±0.8*	30.8±1.0*	37.7±0.6*	33±0.8*	53.5±1.0
*B. anthina* FC0974	26±1.4	24.5±1.3	23.5±0.6	24.5±0.7	30.5±1.3	29±1.4	52.0±1.2
FC0974 nonmucoid	26.5±1.3	30.8±1.7*	23.8±1.3	33±1.7*	31.8±0.5	29.7±0.6	55.8±1.1*
*B. ambifaria* CEP0958	20±0.8	30.8±1	25.5±0.7	28.5±0.7	26±0.8	32±1.4	50.0±0.7
CEP0958 nonmucoid	23.5±0.6*	33.5±1.3*	29±1.4*	0±0*	15.3±0.5*	35.5±0.6*	54.6±0.9*
*B. stabilis* LMG18888	20±0.8	27.5±0.6	24.5±0.6	29.5±2.1	28.8±1.9	31.5±0.7	54.8±0.4
LMG18888 nonmucoid	19.5±0.6	30.3±1.0*	22.5±0.6*	15.7±0.6*	31±1.4	31±1.4	57.4±1.5*

Susceptibility was evaluated after 24 h incubation at 37°C by measuring the diameter of the cell growth inhibition zone. The data are means ± SD from the results of at least three independent experiments. A *P*-value of <0.05 was considered significant compared to the respective parental isolate (*).

Oxidative stress induced by the presence of H_2_O_2_ was also evaluated for each pair of mucoid/nonmucoid variants. Despite the high susceptibility of all mucoid and nonmucoid variants to this compound, the nonmucoid variants of *B. anthina* FC0974, *B. ambifaria* CEP0958 and *B. stabilis* LMG18888 were more susceptible than the mucoid parental isolates. Contrastingly, the nonmucoid variant of *B. multivorans* D2095 was more resistant than the mucoid parental isolate (Table 2).

### Nonmucoid variants showed increased biofilm formation ability but no difference in siderophore production

**Figure 6:**
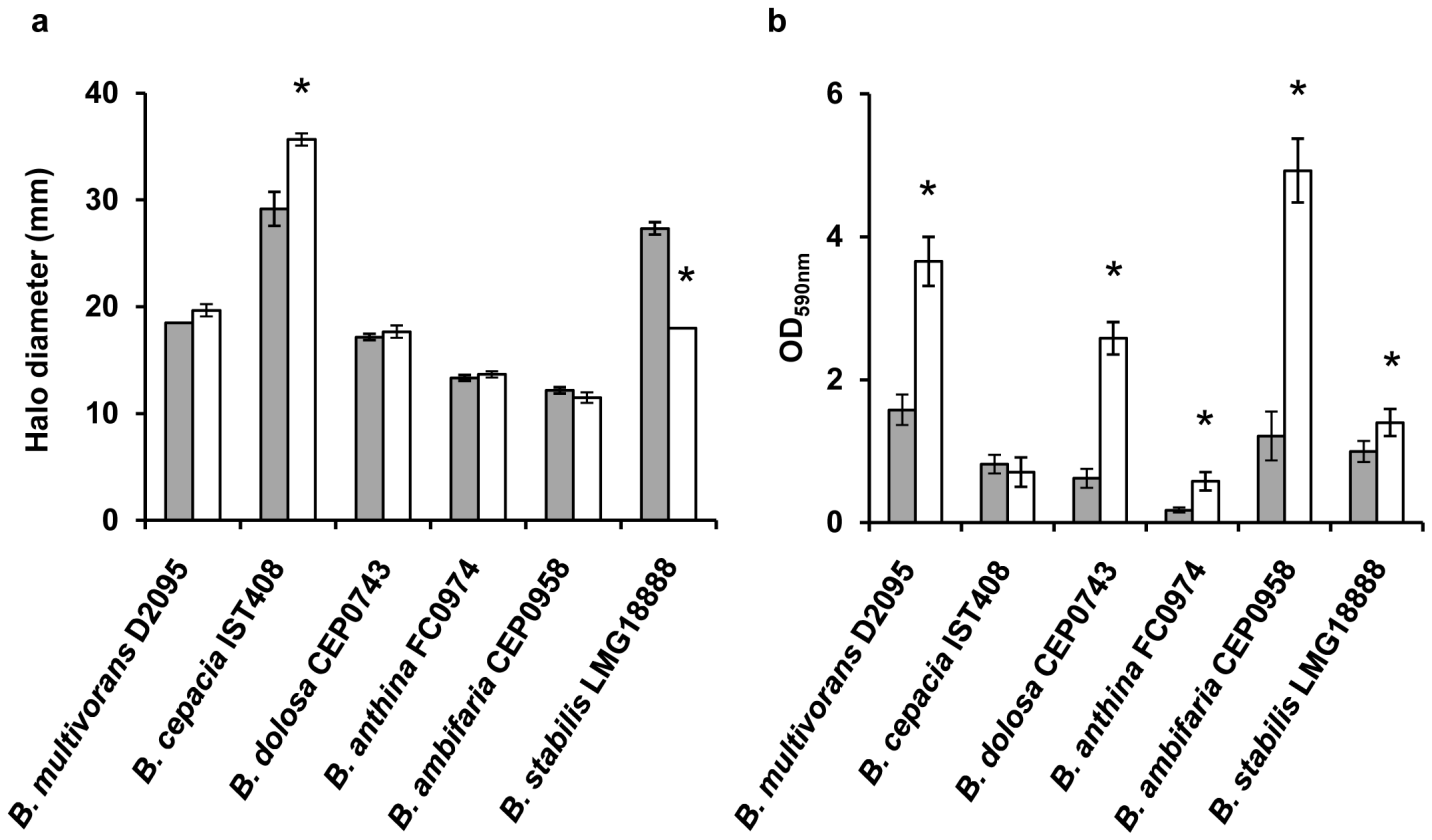
Production of siderophores and biofilm formation by the mucoid isolates (grey bars) and nonmucoid variants (white bars). (a) Siderophore production was evaluated after 48 hrs at 37°C in CAS-MM medium. (b) The biomass of the biofilm was assessed after 48 hrs incubation at 37°C in polystyrene microtitre plates containing SM medium. The data are means ± SD from the results of at least three independent experiments. A *P*-value of <0.05 was considered significant compared with the respective mucoid parental isolate (*).

Since siderophore and biofilm formation have been reported as important virulence factors in *Bc*c bacteria, their production by the mucoid and nonmucoid variants was assessed. As regards siderophore production in MM-CAS medium, the only differences detected were for the mucoid and nonmucoid variant pairs of *B. cepacia* IST408 and *B. stabilis* LMG18888. Effectively, the nonmucoid variant of IST408 produced an higher amount of siderophores when compared to the mucoid parental strain. In the case of *B. stabilis* LMG18888, the nonmucoid variant produced lower amount of siderophores (Fig. 6a).

Biofilm formation, as measured by crystal violet staining, was increased in all nonmucoid variants compared to the respective parental mucoid isolate. *B. cepacia* IST408 was an exception, since no significant differences in biofilm formation ability was detected for the mucoid and nonmucoid variant (Fig. 6b). These results are in agreement with previous observations showing that *B. multivorans* D2214 nonmucoid clinical isolate form thicker biofilms in vitro when compared to the mucoid clonal D2095 isolate [13].

## Discussion

Conversion to the mucoid phenotype is well known in *Pseudomonas aeruginosa*, with the initial isolates colonizing CF patients airways being nonmucoid, but becoming mucoid during the course of respiratory chronic infections due to overproduction of alginate [25]. An opposite observation was reported for other CF opportunistic pathogens, namely for *Bc*c bacteria in which morphotype transition is mainly from mucoid-to-nonmucoid [12]. To understand this morphotype transition in *Bc*c, we have previously reported the characterization of a pair of clonal isolates of *B. multivorans* (D2095 and D2214) from a chronically infected CF patient where a mucoid-to-nonmucoid transition had occurred [13]. Compared to the mucoid parental isolate D2095, the nonmucoid isolate D2214 exhibited a reduced expression of some virulence factors, it caused a lower mortality rate of *G. mellonella* larvae, but displayed a higher survival ability under nutrient starvation and increased biofilm formation ability [13]. In this study we have investigated the morphotype stability of the mucoid D2095 and nonmucoid D2214 isolates after exposure to stress conditions. We never observed mucoid morphotype variation when D2095 or D2214 isolates were grown under standard conditions (for instance SM, MM, and YEM liquid media, aerobically at 37°C for 3 days) or by multiple passages onto solid media, confirming their morphotype stability. Nevertheless, during prolonged incubation for at least 7 days, nonmucoid variant colonies arised from the mucoid D2095 culture while no mucoid colonies were ever recovered from nonmucoid D2214 culture even for longer periods of incubation. The nonmucoid variants of D2095 are stable since they maintained the same morphotype in the absence of the stress condition.

Under laboratory conditions, mucoid clinical isolates of *P. aeruginosa* give rise spontaneously to nonmucoid variants, while the emergence of the mucoid phenotype from a nonmucoid isolate has been shown to occur only if induced by stress conditions such as carbon, nitrogen and oxygen limitations and osmotic stress [8]–[10]. In our work we showed that temperatures lower and higher than normal body temperature (22°C and 42°C) combined with prolonged stationary phase were a strong stimulus for morphotype variation. At the temperature of 4°C, where no growth occurs, no variation of the mucoid-to-nonmucoid morphotype was observed, suggesting that only metabolically active cells are prone to variation. High osmolarity and oxidative stress conditions in vitro also triggered mucoid-to-nonmucoid morphotype variation in *B. multivorans* D2095. This shows that the same cues stimulate genotypic/phenotypic variation in different bacterial species.

The analysis of mucoid-to-nonmucoid morphotype variation under stress conditions was extended to other *Bc*c and non-*Bc*c mucoid isolates, with the results showing that this morphotype variation ability is widespread among the genus *Burkholderia*. These results confirm the high adaptability of *Burkholderia* to changing environments and corroborates the findings of Vial and collaborators [26] who have demonstrated phase variation in the clinical isolate *Burkholderia ambifaria* HSJ1. The occurrence of phase variation in this isolate, resulted in two distinct colony morphotypes displaying different phenotypic and virulence properties, with one adapted to the human host and the other to the rhizosphere. In another study, Chantratita and collaborators [27] described seven distinct morphotypes of *Burkholderia pseudomallei* arising during host passages or under different stress conditions including nutrient starvation, iron limitation and growth at 42°C. Type-I, type-II and type-III morphotypes of *B. pseudomallei* were associated with different survival ability under oxidative stress, in the presence of antimicrobial peptides, under anaerobic conditions and within human macrophages [28].

Since CF patients are often exposed to antibiotic therapy, we sought to determine whether the presence of antibiotics of clinical relevance could trigger mucoid morphotype variation. This was previously shown for two *Burkholderia* clinical isolates in the presence of ciprofloxacin and ceftazidime [16]. In addition to the former antibiotics we have also found that amikacin and kanamycin triggered the nonmucoid morphotype variation of *B. multivorans* D2095. Furthermore, ciprofloxacin induced mucoid-to-nonmucoid morphotype variation in three other *Bc*c isolates from different species. Considering that CF patients dosage of inhaled amikacin ranges 1 g day^−1^, intravenous ceftazidime (2–3 g, every 8 hrs) and oral ciprofloxacin (1.5 g day^−1^), perhaps the antibiotics concentrations used in this study are comparable to the concentrations achievable in the lungs. Our observation raises the question on whether antimicrobial therapy, by possibly triggering morphotype variation within the CF lung, contributes to disease progression. Further studies are needed to address this question.

We have also investigated whether the nonmucoid morphotype of the variants (lacking EPS production) affected other cellular properties such as virulence, resistance to several stresses, and biofilm formation. As regards virulence, several studies have highlighted the importance of the EPS and the mucoid phenotype both in vitro and in vivo models of infection. For example, Conway et al. [15] have shown that a mucoid isolate from a CF patient has reduced interaction with immune cells, which may explain the lower clearance from infected mice when compared to a clonal nonmucoid isolate from the same patient. Similarly, mortality was completely absent or strongly reduced in gp91^phox−/−^ mice challenged with EPS defective mutants [29]. In our work, virulence assays were performed in *G. mellonella*, as this insect has an innate immune system that shares a high degree of structural and functional homology to the innate immune systems of mammals, making it a good model for virulence assessment [23]. Overall, our experiments using *G. mellonella* as infection model showed that the majority of the nonmucoid variants exhibited virulence attenuation. This is in agreement with the role of the EPS as protecting bacterial cells from the host immune system and from harmfull compounds, as suggested by others [15], [30]–[32]. Opposite to these observations on the contribution of the EPS to virulence is the work of Zlosnik et al. [16] showing that CF patients chronically colonized exclusively with nonmucoid bacteria had the most dramatic decline in lung function, followed by the ones colonized with a mix of mucoid and nonmucoid morphotypes. To explain these differences on the role of the mucoid phenotype in virulence in animal models and in humans we may have to consider other phenotypic properties of the mucoid and nonmucoid isolates such as the biofilm formation ability. Previous work with two sequential pairs of *Bc*c isolates from two patients with CF, reported the increased biofilm production in the nonmucoid variant [13], [15]. In agreement with these observations we have also shown increased biofilm formation of the nonmucoid variants from different *Bc*c isolates. As biofilm formation is highly dependent on the interaction of the bacteria with biotic/abiotic surfaces, perhaps the nonmucoid isolates have a disadvantage while facing host immune cells during an acute infection, but are better adapted to persiste in the CF lung chronic infections. Simultaneously, the nonmucoid isolates may have differences in adhesion/invasion of the host tissues. This would explain the increased lung function deterioration observed in CF patients, but requires further investigation.

Colony morphotype variation in bacteria reflects their adaptation to the surrounding environment, whether it occurs by phase variation or by irreversible mutational events. Since phase variation is a spontaneous and reversible process, it is likely that the mucoid-to-nonmucoid switch occurring in *Burkholderia* exposed to stress conditions results from irreversible mutations. The expression of the *bce* genes directing the biosynthesis of cepacian are down-regulated in the nonmucoid *B. multivorans* D2214 isolate [13] and in one *B. multivorans* D2095 nonmucoid variant obtained under prolonged incubation. On the other hand, no differences were observed in a second variant obtained under the same conditions (our unpublished data). Therefore, we hypothesize that *Burkholderia* strains are endowed with more than one mechanism to generate the nonmucoid morphotype. When cells are exposed to unfavorable conditions, phenotypic and/or genotypic diversity is generated in order to give rise to more adapted cells. It is possible that the nonmucoid morphotype is just one of the outcomes of this diversification. Our data do not allow us to conclude on whether the nonmucoid morphotype is selected under the tested conditions due to some advantage or is a consequence of the genetic diversification of stressed cells. A future strategy to uncover the molecular mechanisms underlying morphotype variation in *Bc*c bacteria will most likely require the analysis of several nonmucoid variants obtained under the same conditions and between different conditions, combined with genomics, transcriptomics and phenomics data.

## Supporting Information

Table S1 Gene-specific primers used for PCR.
(DOCX)
