# Supplementary material for: Stress Conditions Triggering Mucoid Morphotype Variation in Burkholderia Species and Effect on Virulence in Galleria mellonella and Biofilm Formation In Vitro
Source: PLoS One. 2013 Dec 16;8(12):e82522. doi: 10.1371/journal.pone.0082522 (PMC3865030; doi:10.1371/journal.pone.0082522)
Supplement: Table S1 — Gene-specific primers used for PCR. (DOCX) [file pone.0082522.s001.docx]

**Table S1.** Gene-specific primers used for PCR.

| **Gene identification** | **Forward primer** | **Reverse primer** |
| --- | --- | --- |
| Bmul_4804 | TCGCATATGACAGACTCTGTATTG | CCATCTAGATTCAAAAGCTGACGACA |
| Bmul_4809 | GGCCATATGGCAGCACGTACT | ATCTCTAGATGCGCCACACTTCA |
| Bmul_4876 | GCAAGTCCGTCACGTTGAACTGTA | CGGCCGTCTCCATGAATTCCT |
| Bmul_4781 | TTCGCCATACGTCCGTGTACGTT | CCGCGAATCCGTAAGACCAGAAAT |
